# Comparative characteristics of the genetic structure of the Syrian cattle breed compared to Holstein and Aberdeen-Angus breeds

**DOI:** 10.5455/javar.2021.h520

**Published:** 2021-06-27

**Authors:** Mohammad Almohammad Alsalh, Anatoly Bakai, Feyzullah Ramazanovich Feyzullaev, Ferdaus Rafailovna Bakai, Tatyana Viktorovna Lepekhina, Gayane Mkrtchyan, Anna Krovikova, Karina Mekhtieva, Ousama Alhammoud Alyaseen

**Affiliations:** 1 Department of Animal Breeding and Genetics, Federal State Budgetary Educational Institution of Higher Education, Moscow State Academy of Veterinary Medicine and Biotechnology- MVA by K.I. Skryabin, Moscow, Russia; 2 Department of Animal Husbandry, College of Veterinary Medicine, Al Furat University, Deirez-Zor, Syria

**Keywords:** Cattle breed, microsatellites, polymorphism, alleles, genetics, Shami, Syria

## Abstract

**Objectives:**

The objective of this study was to perform a comparative analysis of allelic diversity to reveal population-genetic characteristics of animal breeds, namely Shami (SH), Holstein (HLS), and Aberdeen-Angus (A-A).

**Materials and Methods:**

The genetic materials of SH breed animals represented by wool with hair follicles were collected from 39 SH heads in Syria. Also, genetic materials of HLS breed of American selection (*n* = 55, HLS) and bulls and cows of A-A breed bred at breeding enterprises in Russia (*n* = 30, A-A) were collected. Genetic differences between the cattle groups were studied using 11 microsatellite markers.

**Results:**

The cattle breed in Syria was characterized by high genetic diversity, 107 alleles, while the average number of alleles per microsatellite locus was 9.23, which is significantly higher than that in the animals of HLS (6.18) and A-A (5.00). When analyzing the genetic equilibrium for individual locus in SH breed, a deviation from equilibrium at four loci was revealed: TGLA227, SPS115, TGLA122, and ETH225; at one locus in HLS breed: SPS115, for A-A breed: at two loci, i.e., TGLA122 and ETH225. When assessing the level of genetic consolidation, a deficiency of heterozygotes was observed in two of the three studied breeds: 4.8% for SH and 8.0% for A-A. A slight excess of heterozygotes was found in the HLS breed at the level of 0.2%. The average comparative measurement of genetic variation in different populations value for 11 loci for all breeds was 0.069, which indicates that 93.1% of the total variability is due to the intra-breed diversity, and only 6.9% is due to the differences between breeds.

**Conclusion:**

The analysis of the animals belonging to their breed has shown a 100% genetic consolidation and the compliance of individual animals with the respective breeds. The study of genetic distances, adjusted for small samples, revealed the smallest genetic distance between the SH breed and HLS breed, equaling 0.107. The A-A breed, which has its separate origin and has never been imported into the Syrian Arab Republic, adjoins this cluster as an independent branch. Microsatellites can be used as an essential criterion for assessing the population-genetic characteristics of groups of cattle of various breeds (degree of polymorphism, level of heterozygosity, fixation indices, genetic group membership).

## Introduction

Biodiversity is necessary for the survival of organisms, and this factor is becoming increasingly important and holds a significant priority in modern science relating to the breeding of farm animals [1,2]. The preservation of genetic diversity within the biological population enables us to carry out breeding work and adequately respond to varying challenges and production environments [3-6]. In recent years, the level of innovation in molecular genetics has reached the necessary degree of accessibility for using the results of deoxyribonucleic acid (DNA) polymorphism analysis in the field of studying the genetic diversity and the evolution of species and breeds. The current requirements for breeding also include various aspects of the use of polymorphic DNA markers. Presently, the most valuable markers in population genetic studies of animals are microsatellites [7-10].

The search for candidate genes responsible for the level manifestation of economically advantageous quantitative traits as microsatellite alleles can be inherited linked to alleles of interest genes, is of great importance. The search for a locus of financially beneficial traits is carried out based on analysis of the length of microsatellite DNA fragments using special statistical programs and databases containing information on animal productivity [11-14]. Microsatellites, short tandem repeats, consisting of monomers up to six base pairs and a total length of not more than 100 nucleotide pairs (i.e., monoclonal DNA markers), were first described in 1989 [15].

The knowledge of population genetic structure and variability is critical for creating efficient strategies to enhance the productivity, performance traits, conservation, and innovative management of farm animal genetic resources [16].

Genetic markers allow one to obtain information on the characteristics of breeding material, assess the diversity of the gene pool farm animals, predict changes associated with breeding factors, identify potentially highly productive males at an early age, trying to make pairs for genotype selection for a heterogeneous effect in their offspring [17,18]. Molecular genetic monitoring of populations allows you to control their genetic structure to maintain an optimally balanced complex of alleles and analyze the animal genotype at the genes associated with valuable traits. These genes belong to quantitative traits of locus [19,20].

## Materials and Methods

To characterize the studied cattle breed in Syria in the context of the global gene pool of *Bos taurus *cattle, we conducted their comparative study along with the largest world breeds of dairy and meat production sectors, i.e., Holstein (HLS) and the Aberdeen-Angus (A-A) breeds (SIR_SH 39 heads, HLS 55 heads, A-A 30 heads). Molecular markers (microsatellites) were used in this study. DNA was purified using the S-Sorb DNA kit (Syntol, Moscow, Russia,) according to the manufacturer‘s recommendation with some modifications. To study the allelic pool of Syrian cattle of the Shami (SH ) breed along with HLS and A-A breeds, 11 microsatellite locus have been used; these are TGLA227, BM2113, TGLA53, ETH10, SPS115, ETH225, BM1824, TGLA112, TGLA126, INRA23, and BM1818. The products of multiplex amplification were visualized using an ABI3130xl capillary laser analyzer (Applied Biosystems, Thermo Fisher Scientific, Waltham, MA), using the software Gene Mapper, v.4. The statistical processing was performed using the GenAlEx 6.0 [21]. We used the equipment of the center for the collective use of scientific equipment “Biological Resources and Bioengineering of Farm Livestock” of the Federal State Budgetary Scientific Institution Federal Scientific Center “Ernst All-Russian Institute of Animal Breeding.”

## Results and Discussion

### Comparative analysis of the incidence of alleles

The analysis of the genetic diversity about the total number of alleles per locus and the number of effective alleles (Ne) per microsatellite locus in the compared breeds are presented in Table 1. According to this data, the studied animals of the Syrian SH breeds were characterized by the highest level of genetic diversity among the studied breeds: the average number of alleles per microsatellite locus was 9.23, compared with HLS (6.18) and A–A (5.00) animals, and the average for all studied animals was 6.97 alleles per locus (Table 1).

### Analysis of the incidence of private alleles

The revealed number of private alleles, which was nine times greater, indicates a unique diversity of SH cattle breed in the Syrian allelic pool. The origin of studied regional populations probably involved the animals of the Oksh breed, which is assigned, judging by their origin, to hybrid cattle *B. taurus / Bos indicus*, which causes such a high number of private alleles. It should be noted that, for the most part, the private alleles found in SIR_SH were found with a low incidence below or equal to 5% and do not significantly affect the population development. For significant over 5%, six alleles were found in Syrian cattle—at the TGLA53 locus: alleles 150 and 152 with incidences of 8.6% and 11.4%; SPS115: allele 250 with an incidence of 16.7%; TGLA122: allele 141 with an incidence of 7.7%; ETH225: alleles 136 and 138 with incidences of 6.4% and 30.8%. In the HLS breed three alleles—in locus TGLA53: alleles 176 and 188 with incidences of 13.6% and 10.0%; TGLA122: allele 183 with an incidence of 14.5%; and for A-A two alleles—in locus TGLA122: allele 155 with an incidence of 11.7%; and TGLA126: allele 113 with an incidence of 12.5%. The results of the analysis of preservation of genetic equilibrium at individual MS locus are summarized in Table 2 as follows from the data shown in Table 2. A deviation from equilibrium at four loci is revealed in the SH breed: TGLA227, SPS115, TGLA122, and ETH225. For the HLS breed, there was a deviation from equilibrium according to Hardy–Weinberg at one locus: SPS115, in A-A—at two loci TGLA122 and ETH225, with different levels of confidence. The deviation from equilibrium should be considered an indication of an inevitable inbreeding of the studied groups, which is apparently due to the specificity of the analyzed groups. 

### Comparative characteristics of population genetic parameters

A comparison of the observed and the expected levels of heterozygosity makes it possible to evaluate the genetic diversity in a particular sample population breed [9,22]. It is believed that in populations where inbred crosses are possible, the expected heterozygosity (He )does not reflect the actual degree of genetic variation. To assess the variability of a population precisely, a criterion is used, which makes it possible to evaluate the He by determining the level of allelic diversity and its relation to observed heterozygosity (Ho) [23]. In this regard, we compared the actual and the predicted degree of heterozygosity at 11 microsatellite locus (Table 3).

According to the data in Table 3, the degree of heterozygosity of individual locus is different. The contribution to the average level of heterozygosity and the influence of the locus themselves are not the same in the studied cattle breeds. The observed level of heterozygosity ranged from 0.273 at TGLA53 locus in A-A to 0.872 and 0.867 at TGLA227 locus in SIR_SH and A-A, respectively, and up to 0.855 at TGLA53 locus in HLS. The degree of heterozygote deficiency in studied groups of cattle also varied depending on the locus. 

Thus, in the Syrian cattle group, deficiency of heterozygotes was observed in 8 of 11 microsatellite locus and ranged from 3.4% in INRA23 up to 13.6% in SPS115. At locus BM2113, ETH10, and BM1818, the observed heterozygosity surpassed the expected value by 1.3%, 5.3%, and 1.9%, respectively. In the HLSs studied in 6 of the 11 studied locus, the deficiency of heterozygotes ranged from 0.1% at TGLA122 and BM1824 loci to 7.9 and 8.0% at ETH10 and SPS115 locus. In the studied animals of the A-A breed, the deficiency of heterozygotes was observed in 8 out of 11 MSs, which ranged, respectively, from 0.3% at ETH10 locus to 37.1% at ETH225 locus. Analysis for individual breeds (Table 3) shows a deficiency of heterozygotes in two of the three studied breeds: 4.8% among SH and 8.0% among Angus, which indicates a possible inbred population. A little excess of heterozygotes has been found in the HLS breed at the level of 0.2%. The revealed tendencies of heterozygotes deficiency have been observed in the positive *F*_is_ values. The results of the analysis of *F*-statistics indicators are shown in Table 4. The *F*_is_ fixation index enables the determination of the relationship between individual animals and the entire breed in general and can be used to assess inbredness [24,25].

**Table 1.** Average number of alleles and the Ne per MS locus in studied cattle breeds.

Breed	Qty	Average quantity of MS alleles (11 locus)	
		Na	Ne
(SH)	39	9.23 ± 1.14	5.45 ± 0.83
(HLS)	55	6.18 ± 0.40	3.81 ± 0.45
(A-A)	30	5.00 ± 0.36	3.18 ± 0.27
Average	144	6.97 ± 0.54	4.15 ± 0.36

Studied cattle breeds: (SH)—Shami, (HLS)—Holstein, (A-A)—Aberdeen-Angus.

**Table 2.** Analysis of the preservation of the Hardy–Weinberg genetic equilibrium in studied cattle breeds.

Locus	Breed		
	(SH)	(HLS)	(A–A)
TGLA227	133,279**	21,018	13,241
BM2113	26,212	11,094	14,459
TGLA53	154,174	36,420	11,000
ETH10	18,061	6,081	3,458
SPS115	37,343*	26,442*	4,159
TGLA122	109,306*	20,052	18,319*
INRA23	22,538	6,847	6,650
TGLA126	13,266	2,663	10,524
BM1818	24,139	6,775	13,919
ETH225	128,252***	9,461	62,087***
BM1824	8,685	9,632	7,992

Studied cattle breeds: (SH)—Shami, (HLS)—Holstein, (A-A)—Aberdeen-Angus.

* *p *< 0.05,

** *p *< 0.01,

*** *p *< 0.001.

Moreover, the fixation index *F*_is_ value greater than zero indicates a deficiency of heterozygotes in the studied group, and its negative value indicates their excessiveness. The obtained *F*_is_ values show the excess of heterozygotes at TGLA227, INRA23, and TGLA126 locus and its total average value for studied animals at the level of 5.8%. The *F*_it_ fixation index shows the relationship between individual animals and all studied animals in general. The *F*_it_ index and *F*_is_ make it possible to identify deviations from the theoretically expected incidence of heterozygotes in the studied group of animals. The calculation of the *F*_it_ value has shown a 12.2% deficiency of heterozygotes in studied breeds. The average *F*_st_ value for 11 locus for all the studied groups was 0.069, and this means that 93.1% of all variance is due to intra-breed differences and 6.9% due to the differentiation between breeds. 

**Table 3.** Actual and expected degree of heterozygosity in studied cattle breeds.

Breed	TR locus	Degree of heterozygosity		Excess (+)/deficiency (-) of heterozygotes, %	UHe	*F*is
		Actual *H*_o_	Estimated*H*_e_			
(SH)	TGLA227	0.872	0.904	–0.032	0.916	0.036
	BM2113	0.769	0.756	0.013	0.766	–0.018
	TGLA53	0.800	0.902	–0.102	0.915	0.113
	ETH10	0.769	0.716	0.053	0.725	–0.074
	SPS115	0.538	0.674	–0.136	0.683	0.201
	TGLA122	0.744	0.866	–0.122	0.877	0.141
	INRA23	0.769	0.803	–0.034	0.813	0.042
	TGLA126	0.590	0.664	–0.074	0.672	0.111
	BM1818	0.718	0.699	0.019	0.708	–0.027
	ETH225	0.769	0.837	–0.067	0.847	0.081
	BM1824	0.692	0.736	–0.032	0.746	0.059
	Breed average	0.730 0.028	0.778 0.027	–0.048	0.788 0.027	0.060 0.024
(HLS)	TGLA227	0.818	0.781	0.038	0.788	–0.048
	BM2113	0.764	0.726	0.037	0.733	–0.051
	TGLA53	0.855	0.854	0.001	0.862	–0.001
	ETH10	0.709	0.788	–0.079	0.795	0.100
	SPS115	0.400	0.480	–0.080	0.484	0.166
	TGLA122	0.818	0.819	–0.001	0.827	0.001
	INRA23	0.836	0.741	0.095	0.748	–0.128
	TGLA126	0.636	0.583	0.053	0.589	–0.091
	BM1818	0.582	0.599	–0.017	0.604	0.028
	ETH225	0.655	0.681	–0.026	0.687	0.038
	BM1824	0.636	0.638	–0.001	0.643	0.002
	Breed average	0.701 0.041	0.699 0.035	0.002	0.705 0.035	0.001 0.025
(A–A)	TGLA227	0.867	0.803	0.063	0.817	–0.079
	BM2113	0.700	0.774	–0.074	0.787	0.095
	TGLA53	0.273	0.607	–0.335	0.636	0.551
	ETH10	0.667	0.669	–0.003	0.681	0.004
	SPS115	0.633	0.688	–0.055	0.700	0.080
	TGLA122	0.500	0.604	–0.104	0.615	0.173
	INRA23	0.621	0.569	0.052	0.579	–0.091
	TGLA126	0.875	0.658	0.217	0.679	–0.329
	BM1818	0.467	0.641	–0.174	0.651	0.271
	ETH225	0.367	0.738	–0.371	0.750	0.503
	BM1824	0.467	0.563	–0.096	0.572	0.171
	Breed average	0.585 0.058	0.665 0.024	–0.080	0.679 0.024	0.123 0.078

Studied cattle breeds: (SH)—Shami, (HLS)—Holstein, (A-A)—Aberdeen-Angus.

### Identification of genealogical relations between the studied groups of cattle

**Figure 1.**
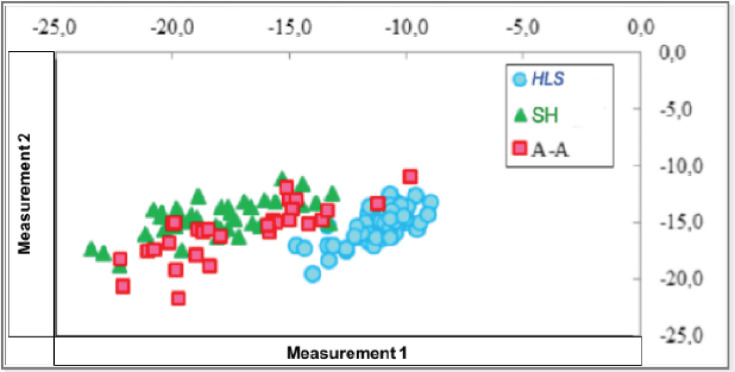
Distribution of studied breeds by reference to their own population. Note: studied cattle breeds: (SH)—Shami, (HLS)—Holstein, (A-A)—Aberdeen-Angus.

The analysis of animals belonging to their own breed to the studied group using microsatellite markers has shown their 100% genetic consolidation. Thus, the studied animals are genetically consistent with their own breeds (Fig. 1). 

The animals of each breed belong to the mentioned populations with 100.0% reliability. It should be noted that the SH and A-A breeds generate clusters overlapping more tightly, while the HLS breed forms its own quite isolated cluster. The genetic distances between the studied cattle breeds, calculated according to Nei [12], are presented in Table 5. The results are supported by Mastrangelo et al. [26].

The smallest genetic distances were found between the SH breed (Syria) and the HLS breed (USA) at the level of 0.107, which is graphically displayed as a combination of these groups in a shared cluster. On the other hand, the animals of the A-A breed, having different genetic characteristics, form a separate branch (Table 5).

**Table 4.** *F*-statistic indicators for studied cattle breeds.

Locus	Indicators		
	*F*_is_	*F*_it_	*F*_st_
TGLA227	–0.028	0.006	0.033
BM2113	0.010	0.063	0.053
TGLA53	0.184	0.271	0.107
ETH10	0.013	0.090	0.079
SPS115	0.147	0.195	0.057
TGLA122	0.099	0.176	0.085
INRA23	–0.054	0.048	0.097
TGLA126	–0.103	–0.058	0.041
BM1818	0.089	0.129	0.044
ETH225	0.206	0.268	0.079
BM1824	0.073	0.154	0.088
Averaged	0.058 ± 0.030	0.122 ± 0.031	0.069 ± 0.007

**Table 5.** Genetic distances between studied cattle breeds.

Breed	Genetic distances as per Nei		
	(HLS)	(SH)	(A–A)
(HLS)	*		
(SH)	0.107	*	
(A–A)	0.405	0.336	*

Studied cattle breeds: (SH)—Shami, (HLS)—Holstein, (A-A)—Aberdeen-Angus. * indicating the mating point between the same breed.

## Conclusion

The study reports a first genetic within breed diversity estimate of the SH cattle population through microsatellite markers recommended by the a Food and Agriculture Organization. Values observed in the present study indicate that the markers used are highly informative for the genetic characterization of SH cattle and give reliable information on genetic diversity and population structure. The study proved that the cattle breed in Syria was characterized by a high level of genetic, which is significantly higher than that in the HLS and A-A breed animals. The genetic distances, adjusted for small samples, the smallest genetic distance between the SH breed and the HLS breed. A-A breed, which has its separate origin and has never been imported into the Syrian Arab Republic, adjoins this cluster as an independent branch. It should be noted that the SH and A-A breeds generate clusters overlapping more tightly, while the HLS breed forms its own quite isolated cluster.

## List of abbreviations

Quantitative trait locus (QTL); total number of alleles (Na); number of effective alleles (Ne); fixation index (*F*_is_); observed heterozygosity (Ho); expected heterozygosity (He); Shami (SH); Holstein (HLS); Aberdeen-Angus (A-A); the deficiency or excess of average heterozygotes (*F*_it_); comparative measurement of genetic variation in different populations (*F*_st_); Syrian Shami (SIR-SH); microsatellites (MS); locus microsatellite (INRA23).
